# Perspectives of policy-makers and stakeholders about health care waste management in community-based care in South Africa: a qualitative study

**DOI:** 10.1186/s12913-017-2236-x

**Published:** 2017-04-19

**Authors:** Lydia Hangulu, Olagoke Akintola

**Affiliations:** Discipline of Psychology, Howard College Campus, University of KwaZulu-Natal, MTB Ground Floor, 4041 Durban, South Africa

**Keywords:** Community-based care, Health care waste, Health care waste management

## Abstract

**Background:**

In South Africa, a new primary health care (PHC) re-engineering initiative aims to scale up the provision of community-based care (CBC). A central element in this initiative is the use of outreach teams comprising nurses and community health workers to provide care to the largely poor and marginalised communities across the country. The provision of care will inevitably lead to an increase in the amount of health care waste (HCW) generated in homes and suggests the need to pay more attention to the HCW that emanates from homes where there is care of a patient. CBC in South Africa is guided by the home-based care policy. However, this policy does not deal with issues about how HCW should be managed in CBC. This study sought to explore health care waste management (HCWM) in CBC in South Africa from the policy-makers’ and stakeholders’ perspective.

**Methods:**

Semi-structured interviews were conducted with 9 policy-makers and 21 stakeholders working in 29 communities in Durban, South Africa. Interviews were conducted in English; were guided by an interview guide with open-ended questions. Data was analysed thematically.

**Results:**

The Durban Solid waste (DSW) unit of the eThekwini municipality is responsible for overseeing all waste management programmes in communities. Lack of segregation of waste and illegal dumping of waste were the main barriers to proper management practices of HCW at household level while at the municipal level, corrupt tender processes and inadequate funding for waste management programmes were identified as the main barriers. In order to address these issues, all the policy-makers and stakeholders have taken steps to collaborate and develop education awareness programmes. They also liaise with various government offices to provide resources aimed at waste management programmes.

**Conclusions:**

HCW is generated in CBC and it is poorly managed and treated as domestic waste. With the rollout of the new primary health care model, there is a greater need to consider HCWM in CBC. There is need for the Department of Health to work together with the municipality to ensure that they devise measures that will help to deal with improper HCWM in the communities.

**Electronic supplementary material:**

The online version of this article (doi:10.1186/s12913-017-2236-x) contains supplementary material, which is available to authorized users.

## Background

Following the Alma Ata Declaration on Primary Health Care in 1978, many low- and middle-income countries (LMICs) have made it a policy priority to shift the care of chronically ill patients from hospitals to the community [1]. The World Health Organization (WHO) has also promoted home and community-based care (CBC) and the concept of task-shifting to deal with health worker shortages in LMICs [2]. In recent years, considerable increases in the funding for HIV/AIDS/TB and the need to meet the millennium development goals have led to a renewed focus on CBC in many LMICs [1, 2].

In sub-Saharan Africa, community-based organisations (CBOs) are a key element in the provision of primary health care services in poor and marginalised communities [2–4]. In the HIV/AIDS sector for example, CBOs often provide care and resources to marginalised populations like sex workers, drug users, gay men, the aged, the poor and the homeless [2, 5]. CBOs are relevant in providing health care because they understand their local communities and they are linked to the populations that they serve [6]. CBOs serve as a link between the health care system, decision makers, and stakeholders in developing health policies and programmes [7]. They are also involved in research development that aims at informing policy [8] and help to facilitate the involvement of communities in planning and implementation of health care in order to achieve ‘health for all’, a key principle for primary health care [5].

Community-based care in South Africa is guided by the home and community-based care policy that was developed in 2001 which is still a draft document. The main thrust of the policy is the provision of CBC in the homes of the patients. The policy encourages community members to participate in the provision of care to the ill people [9]. However, this policy does not deal with how health care waste (HCW) should be managed in CBC. The WHO defines HCW as all waste that is generated in health care facilities, research centres, and laboratories that are related to medical procedures. It also includes waste produced from health care activities in minor and scattered sources including in homes where there is recuperative care, self-administration of insulin and dialysis [10]. HCW management (HCWM) involves segregation, collection, storage, treatment, transportation, safe disposal [11] and monitoring of these activities [10]. When HCW is not properly managed, it could transmit infectious diseases such as HIV/AIDS, hepatitis B and C and tuberculosis to the public, and could cause death [12, 13]. HCW could also reduce environmental aesthetics [14], cause social contagion [15] and also cause the breeding of disease-causing vectors such as cockroaches, flies and rodents [16, 17].

In South Africa, a new primary health care (PHC) re-engineering initiative aims to scale-up the provision of CBC. A central element in this initiative is the use of outreach teams, comprising nurses and community health workers, to provide care to the largely poor and marginalised communities across the country [18, 19]. One would expect that the scale-up of the provision of community-based care will inevitably lead to an increase in the amount of HCW generated in homes and this suggests the need to pay more attention to the HCW that emanates from homes where there is care for patients [20].

In KwaZulu-Natal province where this study was conducted, some challenges with HCWM have been documented. For example, a study was conducted in 30 clinics in iLembe health district, the findings of which revealed that HCW was frequently not segregated from the point of generation to the point of disposal; it was sometimes transported together with goods and passengers, and the vehicles were driven by people who are untrained, unequipped and not registered to handle HCW [21]. Given the recent policy direction of the department of health to promote home and community-based care on a national scale, the perspectives of policy-makers and stakeholders could help shed light on particular issues relevant for policy decision-making on health care waste management in community-based care. Regrettably, little is to be found about the perspectives of policy-makers and stakeholders regarding HCWM in community-based care in South Africa. In this study, we sought to answer the following questions: What are policy-makers’ and the stakeholders’ perceptions regarding HCWM in community-based care? How do policy-makers and stakeholders describe challenges related to HCWM in CBC? How do policy-makers and stakeholders address the challenges related to HCWM in CBC?

## Methods

### Research design

This was a descriptive qualitative study [22] that helped to provide in-depth insights into policy-makers’ and stakeholders’ perceived challenges with HCWM, their causes as well as descriptions of how the challenges related to HCWM were addressed.

### Study setting and context

This study was conducted in 29 resource-scarce communities located on the outskirts of Durban, KwaZulu-Natal, South Africa. Of these, 21 were peri-urban communities. Peri-urban communities are segregated communities that were created by the apartheid government in the 1950s and 1960s and were racially structured to stabilize black labour in the industrial economy. These communities are characterised by the presence of small sized houses named after the reconstruction and development programme (RDP) that was initiated by the government in 1994 to promote service delivery. The RDP houses are for the poor who earn less than R3500 per month [23]. Currently, because the government provides low subsidies for developing these houses, RDP houses are usually built on cheap land located away from economic opportunities. The minibus taxi industry provides community members with transport which links dwellers to the cities to access economic opportunities. Because most people in these communities do not work and/or have an unsteady income, they tend to build ‘back rooms’ which are extensions of the main house. They rent the backrooms out to people who are still waiting for RDP houses as a way of earning a living. Some households rent out the RDP houses and opt to live in the backrooms [24].

Furthermore, three of the communities that were included in the study were informal settlements. Informal settlements consist of houses that are illegally built on private land, government owned land or tribal land. People who live in informal settlements travel from various places such as rural areas or peri-urban communities and some are foreign nationals who are in search of formal housing and employment. Informal settlements have a high rate of unemployment, food insecurity and poverty [25, 26].

Five were rural communities: these are areas that are neither peri-urban nor informal settlements. The communities are settlements usually located far from economic centres and affordable transport is limited and expensive. They are occupied mainly by the older populations that have retired and rely on subsistence agriculture, social grants and allowances from family members who work in cities [27]. All the 29 poor resource communities are characterized by high rates of unemployment and poverty; there is a lack of quality social services such as education, health and transport services. Municipal services such as water, sanitation and electricity are basic and free [28–30]. These communities are serviced by the eThekwini Municipality of KwaZulu-Natal.

### Study participants

Four kinds of participants were included in our sample: nine ward councillors who are policy makers, five area cleansing officers, ten managers of CBOs and six education officers who are stakeholders in charge of overseeing general waste management activities in the communities. The number of years of experience in the community ranged from one to 13 years as described in Table 1 below.

**Table 1:** Roles and demographic characteristics of policymakers and stakeholders

Post of the official	Role in the community	Total number of participants	Range of years of service of participants
Ward councillors	These are policy makers who are employed by the government at the municipality level. They are community representatives who provide leadership and guidance to the community and facilitate communication between the community and the government at the municipality level.	9	4–6
Area cleansing officers	These are stakeholders and are government employees at the municipal level. They supervise waste management contractors, inspect communities to ensure that waste is collected and they oversee garbage bag distribution within the communities.	5	4–5
CBO managers	These are stakeholders who manage non-profit organisations that provide community-based care programmes in the communities.	10	8–13
Education officers	They are stakeholders who are employed by the government at the municipal level. They develop and facilitate education programmes on waste management in the communities.	6	1–3

### Sampling procedure for the participants

CBO managers were selected using snowball sampling. We contacted two CBO managers known from previous research. These managers provided contact details of the other managers that they knew. From the contact details provided, eight CBO managers from different communities were recruited purposively if their organisations offered home-based care services. CBO managers were included in the study because they oversee CBC programmes that are responsible for generating health care waste. The CBO managers were chosen if they were willing to participate in the study. Ten CBO managers (one per organisation) participated in the study while three were not available during the study period. Contact details of the ward councillors, area cleansing officers and education officers who served the 29 communities were obtained from CBO managers. The ward councillors, area cleansing officers and education officers were chosen if they were willing to participate in the study and if the CBOs fell within their jurisdiction. Ten ward councillors served the thirteen communities. However, only nine of them participated in the study, the remaining one declined to participate in the study citing a lack of interest. Five area cleansing officers and six education officers participated in the study because the thirteen communities fell within areas of their jurisdiction. All participants were selected if they worked for a period of six months or more because respondents with such length of work experience were in a better position to provide insights to the study.

### Ethical considerations and data collection procedure

Ethical approval for this study was obtained from the Humanities and Social Science Research Ethics Committee of the University of KwaZulu-Natal, South Africa. Semi-structured interviews were conducted with nine policy-makers and twenty-one stakeholders and these were guided by interview schedules. In order to develop the interview schedules, we conducted a rapid review of the literature on community-based care and health care waste management in South Africa. Information derived from this review was then used to develop open-ended questions for the interview schedules for each group of participants (see the Additional file 1). The interviews were conducted by the lead author. The interview schedule covered three main themes: 1) the policy-makers’ and the stakeholders’ perspectives regarding health care waste management in community-based care 2) the policy-makers’ and stakeholders’ perceived challenges regarding health care waste management in community-based care 3) strategies employed by policy-makers and stakeholders to address the challenges related to health care waste management in community-based care. Participation in the study was voluntary and anonymity was achieved through the use of titles and not names. The objectives of the study were explained to the participants; informed consent was sought and all participants gave both written and verbal informed consent. Permission to record all interviews was sought and granted. All interviews were conducted in English in the participants’ offices and they lasted from about 40 to 60 min. Data collection took place from August 2014 to March 2015. Permission to publish the findings was sought and granted both from the ethics committee and the participants.

### Data analysis

All the recorded data was transcribed verbatim in English by the research assistant. Data analysis was conducted using the six steps of thematic analysis suggested by Braun and Clarke [31]. The first step involved familiarization with data through reading all the transcribed scripts. We immersed ourselves in the data in order to familiarise ourselves with it. In the second stage, we identified and generated themes. In the third stage, we re-read all transcripts identified and generated all codes. In the fourth stage, we generated themes from the codes. Fifth, we read and grouped the identified themes and then proceeded to identify sub-themes. We discussed each of the themes and sub-themes. We reached consensus as such all the main themes and sub-themes are presented in the findings.

## Results

The following themes were derived from the data: Perceived HCWM practices in community-based care by policy-makers and stakeholders, the perceived challenges, the perceived causes of the challenges and strategies used to address the challenges of HCWM practices in community-based care by policy-makers and stakeholders. All the major themes are in bold while minor themes are italicised in bold. Narratives showing the opinions and positions of participants within their specific roles are presented under each theme and subthemes. However, a few dissenting opinions were also noted when comparing opinions and positions among different roles. Such dissenting opinions have been presented as such in the narratives together with verbatim quotes.

### The perceived health care waste management practices in community-based care

Participants explained that the Durban Solid Waste (DSW) unit of the eThekwini Municipality is responsible for overseeing all waste management programmes in their communities. Waste management services are free for those in rural, peri-urban communities and informal settlements, because they are subsidised by the government. All participants indicated that health care waste is mixed, treated as domestic waste and is removed together with domestic waste from all homes. They further explained that, DSW has garbage trucks and waste collectors that remove all domestic waste which is mixed with health care waste from suburbs. The ward councillors indicated that, as a way of empowering communities, the municipality awards tenders to community members. The selected community members work as waste management contractors whose job is to remove all waste from homes to the disposal sites. Ward councillors and area cleansing officers indicated that all tenders are advertised in the media and the most competent contractors are offered the tenders. Contractors sign contracts with DSW and they are given rules and regulations on how they should operate:

*“Yes they sign a contract document that binds them on how to work. It is a very thick document which constitutes what they are supposed to do and how and what is expected of them and their staff.” (Area Cleansing Officer 1).*

All participants were asked to give an account of how HCW is managed in the communities that they served. The CBO managers explained that they advised their community health workers (CHWs) who provide home visits to the patients to dispose of the HCW in black garbage bags or in any other plastic bag and to tie the plastics containing HCW to prevent spillage. Contrary to what the CBO managers said, most area cleansing officers were defensive when they were asked to explain how HCW is managed and removed from homes in the communities that they served. Most of them indicated that they were not aware that HCW that was generated in homes:

*“The thing is we do not know that there is a problem like that, if we knew of a house that has a patient, then maybe we can make an arrangement.”* (Area Cleansing Officer 5).

Area cleansing officers emphasised that there is a private company responsible for removing HCW from hospitals and clinics, yet they did not say who is responsible for removing HCW from homes where patients are receiving care. They insisted that their main role is to ensure that all domestic waste and not HCW, generated in homes is removed by community waste management contractors:

*“A private company collects all the waste for the hospitals and the clinics, but us in the DSW unit we only collect domestic waste.”* (Area Cleansing Officer 2).

Only two area cleansing officers and all education officers were willing to openly discuss the issue of HCWM. The two acknowledged that they are aware that HCW is generated in homes and is usually treated and removed together with domestic waste. The two area cleansing officers explained that they handle HCW as domestic waste because it is not in large quantities unlike at the hospitals. One of them said:

“*Such cases are few that we have health care waste… so because it may be only one residence that has a patient, we encourage such people to put everything (HCW) in a plastic bag and tie it up, then place it in the house bin, because there is no other way. Unless if there is a lot of people, then we can refer them to those that deal with medical waste in the clinics and the hospitals, they have their own special truck that collects medical waste.”* (Area Cleansing Officer 3).

All participants were asked to describe the challenges related to HCWM in the communities that they served as well as their perspectives about the causes of these challenges. The challenges are discussed at the household/community and the municipal levels. At the household level, the main themes that emerged are lack of segregation of waste by households and illegal dumping and these are discussed in detail below.

### The perceived challenges with health care waste management practices in community-based care at household level

This theme will discuss the challenges that impede health care waste management practices in CBC, the causes and the strategies used to deal with the challenges. The themes are presented at the community level and municipality level. At the community level, challenges range from lack of segregation of HCW by households to illegal dumping. At the municipality level the challenges range from corrupt tender processes to inadequate funding for general waste management. A wide range of causes of the challenges and strategies used to deal with the challenges are provided and all themes and sub-themes are summarized in Table 2 and will be discussed in detail.

**Table 2:** Summary of the challenges, causes and solutions to the challenges of health care waste management

Sources of the problems	Problems	Causes of the problems	Solution provided to the problems
The community level	1. Lack of segregation of health care waste	oLack of knowledge about segregation of health care waste. oLaziness to take out health care waste on the day of collection oNegative attitudes about waste management. oIrregular collection of health care waste by waste oLack of sufficient garbage bags. oLack of participation of community members regarding waste management programmes. oThe presence of back rooms. oLong distance between the waste storage facilities and homes in the informal settlements. oSlow change in the rural areas	oCollaboration of providing education about waste management in general oLiaising with government for adequate resources
	2. Illegal dumping		
The municipality level	1. Corrupt tender processes		
	2. Inadequate funding for the waste management in general		

### Lack of segregation of waste in homes by households

All participants revealed that generally, waste segregation is a responsibility of the households and that waste collectors are responsible for collecting the waste from homes and transporting it to the landfill. They also explained that households do not separate the HCW and as a result, waste collectors end up collecting and transporting the unsegregated HCW to the landfill. There were incidences of waste collectors being pricked by needles while collecting waste from homes. Participants revealed that, such incidences were investigated and the affected individuals sought medical attention.

*“Another thing is, needles which people use when they have diabetes or anything, they just throw them away. We have had incidences where our workers have been pricked by them because even if you give them gloves a needle is a needle, it goes through. But* s*uch incidences are thoroughly investigated.”* (Education Officer 1).

All participants felt that the possible cause of lack of segregation of waste by the household members was lack of knowledge about waste segregation. They believed that there was a need for community members to be educated on how to handle HCW. One area cleansing officer said:

*“…Communities must be taught to at least wrap a needle with a tissue or something before disposing it…Just for them to learn simple things like that for now.”* (Area Cleansing Officer 5).

### Illegal dumping

All respondents indicated that all the study communities were facing challenges with illegal dumping. Community members disposed of HCW together with domestic waste illegally in the bush, on the roads and in streams.

*“There is litter all around. You go to the roads, rivers and streams you find that they are full of litter. People throw dirty diapers and other things there…” (Ward Councillor 7).*

All participants said that illegal dumps are a hazard to children who make these dumps their playgrounds and to scavenge for used items. One CBO manager said:

*“With these illegal dumps that are right next to our homes. You find that children go to these areas and play there! It is dangerous!”* (Manager C).

Ward councillors believed that illegal dumps created an opportunity for criminal activities especially in peri-urban communities and informal settlements. The councillors said that in these communities, there were instances when they found foetuses at the illegal dumps suspected have been from illegal abortions carried out by young girls in these communities. They also felt that illegal dumpsites were a hiding place for boys who used injectable drugs and disposed of the needles illegally at the illegal dumps. Additionally, one councillor narrated a story where in two separate communities, they found a woman’s body that was burnt in an illegal dump in the bush and in another community, a woman was beaten up and left to die at a dumpsite.

### The perceived causes of challenges with health care waste management practices in community-based care at the household level

All respondents revealed that illegal dumping of HCW was the main cause of the challenges related to health care waste management in the communities. The reasons provided ranged from laziness to lack of space in the communities.

#### Laziness and negative attitudes towards waste management

All participants claimed that community members were illegally dumping HCW because they were too lazy to take it out on the particular days designated for waste collection. They reported that community members disposed of HCW illegally because they believed that this practice was a way of indirectly creating jobs for the waste collectors. This attitude outrage all education officers and the area cleansing officers expressed outrage at this attitude because these practices undermined their work by creating the impression that they are not doing their jobs effectively. One area cleansing officer, with an angry tone, said:

“*The mind-set of the people is terrible! Their attitude towards waste management is unacceptable! Throwing away litter! Anywhere and everywhere! Because they believe that they are ‘creating jobs’! Who does that? Really?* (Area Cleansing Officer 4).

#### Irregular collection of health care waste by contractors

All participants agreed that irregular collection of waste caused the creation of illegal dumps. For example, CBO managers and ward councillors explained that there were several instances when waste was left uncollected from the communities for several days without any notices from the waste contractors. They revealed that the uncollected waste is scattered by animals that tear up the garbage bags to scavenge for food. To ensure that waste management services continue, area cleansing officers seek permission to use the DSW trucks (meant to serve suburbs) to collect all waste from the communities. The education officers and the area cleansing officers revealed that they have the power to fine and penalise the contractors who fail to adhere to the contracts. Those that do not deliver the required services or pay the fines are reported to top management so that their contract can be cancelled and their services terminated.

*“We report those that do not pay the fines and those who continually fail to deliver services according to the stipulated contract. We recommend to the top management that they should not be paid the full amount or their contracts should be cancelled.”* (Area Cleansing Officer 4).

#### Insufficient garbage bags

Education officers and area cleansing officers provided more insights about this issue because they are directly involved with waste management and related issues. They explained that households in peri-urban and rural areas as well as those in informal settlements receive only two garbage bags per week while those in the suburbs received two months’ supply. With regards to the use of garbage bags, the education officers expressed concern. They explained that most households in the suburbs adhere to proper waste management practices and they use the garbage bags for the intended purposes, while in most cases, those in peri-urban, rural areas and informal settlements use the garbage bags for other purposes such as storing clothes, committing crimes such as storing dead bodies or storing foetuses resulting from illegal abortions.

However, the main reason why only two garbage bags were provided is inadequate funding for sufficient supply of garbage bags by the municipality. Most area cleansing officers said that two garbage bags per week were not sufficient to accommodate the HCW that is generated on a daily basis. They said that this was an issue beyond their control and there was nothing they could do to rectify the problem because they work with a given budget which was limited. They also said that they are discussing the issue with their superiors to find a possible solution regarding budget increments. One area cleansing officer said that they negotiated with their superiors in management for several years to offer households at least a three months’ supply of garbage bags without success.

“*There is nothing we can do because it is something we have raised with the management, saying that people should be given a three month supply as it happens with the suburb… They said that they have problems relating to budget and the money is not adequate for buying garbage bags for households…” (*Area Cleansing Officer 2).

#### Lack of participation in waste management programmes

Education officers stated that with the help of community leaders and ward councillors, they organise clean-up campaigns in the communities aimed at removing all illegal dumps. They hold workshops with community members and teach them about the importance of keeping the environment clean. During the campaigns, education officers encourage community members to take ownership of the problem (illegal dumping). After that they choose a day for cleaning and removing all the illegal dumps in the communities. Education officers said that they felt disappointed because community members do not commit to such programmes. They indicated that many community members do not show up for clean-up sessions. They believed that such acts undermine their work.

#### Back rooms in peri-urban communities

Area cleansing officers blamed some households for creating enabling environments for illegal dumping in the community. They revealed that some households have illegal backrooms. They said that backrooms are structures that most households build as an extension of their own house in peri-urban communities. Residents rent out these rooms to tenants as a way of earning a living. Area cleansing officers revealed that when such structures are built, no toilets or refuse bags are provided to the tenants, because they are not legal occupants. They said that occupants of such back rooms are also expected to share all the sanitation facilities with the landlords but many of them dump their HCW illegally.

*“The refuse bag distributors know that they should give one plastic bag to each household, but then there are houses with 4 or 5 tenants. Tenants also need refuse bags, but they do not get them because the people who give bags don’t know them, they are not appearing on their database so they are staying illegally.”* (Area Cleansing Officer 1).

All area cleansing officers suggested that government must take responsibility for addressing this problem because it has to do with service delivery and that it is a housing issue that needs to be dealt with by the housing department.

#### Long distance between homes and waste storage facilities

Ward councillors and area cleansing officers revealed that in informal settlements, roads are inaccessible for the waste collectors. As such, waste collection points are built close to the main roads. All households are expected to remove their waste from homes and store it in these facilities on a daily basis. They explained that the long distance between the homes and the waste disposal facilities was a disincentive for community members which negatively affected their use of such facilities. Area cleansing officers said that this issue is beyond their control and felt that it is a service delivery issue that is supposed to be addressed by the government.

#### Slow change in rural areas

This was an issue that was raised by only one education officer. The education officer believed that change in rural areas is slow. Households in rural areas still buried HCW even if they were educated about its negative impacts. In response to this challenge, she said that all education officers continue to offer education about proper management of waste. The education officer also believes that there is a need for the municipality to put extra effort into monitoring waste management activities in these areas.

### Perceived challenges with health care waste management practices at the municipality level

All participants felt that there are challenges at the municipal level that hamper proper management of waste in homes in the communities. They identified corrupt tender processes and insufficient funding for waste management services as problems at the municipal level.

#### Corrupt tender processes

All participants believed that the service delivery issue was not within their purview and is therefore an issue that they could not address. All area cleansing officers expressed disagreement with the process involved when choosing contractors responsible for managing waste in the communities. They felt that the tender process was corrupt and lacked transparency. The area cleansing officers revealed that most contractors got their tenders because they had political connections with the tender board. They complained that the government does not involve area cleansing officers in the selection of the contractors even though they are in a better position to do so because they work directly with the people and are able to know their capabilities. They criticised the process and indicated that this interfered with waste management services in the communities. They observed and believed that the contractors that are offered the tenders are incompetent and unskilled to handle waste in general. They said that some waste contractors used open vans when collecting the waste from the communities:

*“You find that they use open vans and staff in the same vans to collect the waste.”* (Area Cleansing Officer 5).

The education officers revealed that the contractors’ trucks constantly broke down and as a result, waste is left uncollected from communities for several weeks:

*“I won’t lie, there are times when the trucks break down and waste is left uncollected. When we ask them they say they are doing something about it. They delay to replace the trucks.”* (Education Officer 2).

Area cleansing officers and education officers felt the constant breakdown of the contractors’ trucks caused households to resort to illegal dumping. They also said that they have powers to fine and terminate the contracts of the offending contractors. However, area cleansing officers felt that their powers were undermined by the tender board that turned down their recommendations. They indicated that such acts caused conflict between them and the contractors. Area cleansing officers believed that most contractors lost respect for them and undermined their job.

“*Most of the contractors are politically connected. Sometimes you report and recommend that the contractor’s contract should be cancelled because he or she is not performing but you find that they have been rewarded with a tender again. Then we look like we are bad people and contractors cannot respect us anymore, they do what they want, you know! We end up dealing with one problem that is not getting solved.” (*Area Cleansing Officer 4).

#### Inadequate funding for waste management programmes

Ward councillors, area cleansing officers and the education officers believed that generally, all waste management issues were not seen as a priority issue by the government as are issues relating to provision of housing for the people citing insufficient funding towards waste management by the government. Two education officers felt that the municipality was not willing to provide sufficient funds for clean-up sessions because it was not a priority issue to the government.

*“Collection trucks and resources for clean-ups are costly. One of the challenges is funds. There are limited funds for clean-ups.”* (Education Officer 1).

One education officer said that insufficient funding has a negative impact on human resources. He said that the job of an education officer requires more human resources due to the fact that they inspect all communities and also attend meetings. Some meetings were held on the same day and same time, and as such it is hard for them to prioritise where to go because all meetings are important and require their attendance. Even though they are each assigned to attend different meetings, they are still unable to attend all of them.

*“There are 18 meeting rooms and only three of us and the challenge is that sometimes there are multiple meetings on the same day due to a lot of war rooms. We then have to separate ourselves between the war rooms but we cannot make it. There is so much demand and we are few.”* (Education Officer 3).

On the other hand, ward councillors revealed that general waste management issues were not a priority on a list of their community development programmes. They revealed that the top developmental issue is housing followed by unemployment. They also indicated that even community members are not interested in any waste management issues because they are more concerned with housing and employment issues.

*“People are hungry, they want jobs and houses. So when you talk about waste no one will listen they all leave you because they are not interested.”* (Ward Councillor D).

### Strategies used to deal with health care waste management challenges in community-based care combined at the household and municipality levels

All participants indicated that, they do not provide programmes directly related to health care waste management. All programmes that are provided aim at managing waste in general and these strategies are discussed below.

#### Collaboration

Education officers said that they have taken some steps to address the problem of lack of segregation of waste in general, illegal dumping and lack of participation by community members. This includes working with CBO managers, community leaders, ward councillors and area cleansing officers, who said that they collaborate with Departments of Health, Housing, Environmental Affairs and Environmental Health to provide various education programmes to community members. They offer door to door education on general waste management and distribute pamphlets that have information on waste management. They also indicated that they hold monthly ‘*Masakhane road shows’* where the public is educated on the separation of various waste. *Education trucks* (mobile classrooms) are provided on site to schools and organisations, to offer training on waste minimization. *Enviro-forums* are conducted with the business owners, health organisations, community members and councillors that aim at having effective coordination on issues regarding the protection of the environment. *Special days* are set aside to raise issues on the environment and the importance of managing general waste. Weekly *landfill site tours* that cover general waste management topics, financial issues, recycling and conservancy management are conducted. Lastly, *buy back and drop off centres* are advertised*.* These are recycling initiatives where community members can drop of recyclable products in exchange for money at buy back centres and also drop off recyclable products for non-reimbursement at drop off centres.

Education officers also indicated that they hold clean-up sessions. In instances where community members do not show up, they reschedule such sessions and continue to mobilise the community members. They collaborate with the Environmental Health Department and hold workshops with the community members to educate them about the importance of the managing various kinds of waste.

*“We postpone it. We do not just give up at the first point. We call another meeting and we involve the ward councillors and the environmental health department so that they advise the community on the hazards that come with a dirty place.”* (Education Officer 3).

Education officers also encourage people to adopt a spot. This is usually done after cleaning an area that was previously an illegal dump. Various people are encouraged to adopt and own such spots to use them as gardens or a play park. Names of the owner (the adopters) are displayed on those spots and are published in community newspapers. Annual competitions are held and prizes are given to the adopters that manage and sustain the spots. This is a way of encouraging people to participate in the clean-up sessions. They also indicated that they focus more on providing education in schools to target children. They do this with the hope that the children would implement what they learn at school in their homes. They also hoped that the households would learn from the children.

*“What we do is increase the levels of education in schools. So we won’t need a lot of money. Therefore, the more people are aware about proper waste mismanagement, the more they take initiative and the less money spent.” (*Education Officer 3).

#### Reporting and liaising with government

All ward councillors, area cleansing officers and education officers felt that they has no power to address issues about corrupt tender processes. They said that these are issues beyond their control because they are involved with politics. However, they address issues regarding distance between homes and waste storage by reporting the matter to the Department of Human Settlements that is in charge of housing issues. On the other hand, all ward councillors, area cleansing officers and education officers explained that to deal with the insufficient funding for clean-up sessions and for garbage bags, they are still negotiating with the government to increase its budget:

*“We do have meetings where we present all our challenges. So it is in these meetings that we try and negotiate with our superiors that we need resources for waste management, for example they must provide more garbage bags for the households…” (Ward Councillor A).*

## Discussion

Previous studies show that HCW is improperly managed in hospitals and clinic settings [13, 32–36]. Our study provides nuanced qualitative findings, which illustrates that HCW is also not properly managed in CBC. This finding contributes to the body of knowledge on HCWM. The finding that the municipality is in charge of overseeing all domestic waste management in the communities including HCW is consistent with the requirements by South African National Standards (SANS 2004) on HCWM [37]. The SANS 2004 states that, HCW that is generated in homes as a result of care for a patient is assumed to be in small quantities hence SANS (2004) requires municipalities in charge of managing domestic waste to handle, transport and treat this waste before its disposal [37]. However, the findings reveal that, in practice, HCW is treated as domestic waste in contravention of the SANS requirements.

Furthermore, it is intriguing that participants assume that HCW that is generated in homes as a result of care for a patient is in small quantities. Yet, South Africa has the highest HIV prevalence in the world and has about 5.6 million people living with HIV [38] most of whom receive care at home [39]. South Africa also has the largest number of TB incident cases in the world [40]. Given that the standards were developed in the year 2004, it seems reasonable to argue that it does not take into account subsequent policy development that have led to the rise in the home-based care activities in South Africa [2]. These include high prevalence of HIV and TB as well as the recent primary health care re-engineering initiative which aims to scale-up the provision of home health care services to communities across the country through outreach teams [18, 19]. The existing and new policy developments highlight the need for policy-makers to revise the policy on HCWM in CBC.

Area cleansing officers expressed dissenting perspectives about the management of waste in homes. While some claimed that they are not aware that HCW was being generated in homes, others acknowledged that it is mixed with domestic waste. This indicates that HCW from homes is not treated as stipulated in the SANS (2004). Even if the volume of HCW generated in homes is small, this does not diminish the risks that it might pose to the environment and the people. Moreover, this finding shows a misunderstanding about how HCW from homes should be handled by the stakeholders in the municipality. The SANS (2004) [37] requires that all HCW from homes be treated as HCW and not as domestic waste. The standards further require the health care providers who are assigned to the patients to provide containers for storing sharp waste specifically for diabetic patients. As for the other infectious HCW besides sharps, it is recommended that private arrangements with hospitals or clinics should be made for the collection and disposal of HCW from homes by contractors responsible for collecting HCW from hospitals and clinics.

It was clear from our study that health care providers do not provide storage facilities for HCW to the households where there are patients receiving care. Additionally, no private arrangements are made for the collection of HCW from the homes of the patients in CBC. Participants did not seem to know whose responsibility it was to provide these facilities and services. These findings highlight a need for the Department of Health to develop policies that will govern HCW from CBC and other minor sources as is the case with hospitals, clinics and other health facilities. Further, the Department of Health and the Durban solid waste unit (DSW) should develop formal partnerships that will help delineate responsibilities relating to the provision of storage facilities for HCW and the disposal of these facilities.

Stakeholders in this study indicated that separation of HCW in homes is the responsibility of households. Mixing of HCW with domestic waste makes treatment of such waste difficult [36]. Improper segregation of HCW exposes family members to injuries resulting from sharp waste and exposes them to infections [41]. Although education officers indicated that they provide education and awareness programmes to community members in the communities, it is clear from our findings that this has not yielded the desired results because it focuses more on domestic waste not HCWM. There is therefore a need for the Department of Health to work with the area cleansing officers to develop mechanisms for identifying and providing households that have patients with HCW storage bins as recommended by the SANS 2004 [37]. There must also be mechanisms put in place to monitor HCWM activities in homes to ensure compliance.

From the policy-makers’ perspectives, the main reason for illegal dumping by community members is the lack of sufficient allocation of budgets for HCWM which results in shortages in the supply of garbage bags specifically for domestic waste. The area cleansing officers stated that they are in constant negotiation with their superiors for adequate allocation of budget. We found that most households are poor and rely solely on government to provide them with houses and basic services including waste management services. As a consequence, some households in peri-urban communities build backrooms to generate income. Occupants of the backrooms are illegal occupants and they contribute to the problem of illegal dumping of HCW which could cause air, land and water pollution [42]. There is a need for the Department of Housing to develop and tighten enforceable housing laws to prohibit building of illegal structures. Steps must also be taken to deter defaulters.

Furthermore, irregular collection of waste by waste collectors was a major factor contributing to illegal dumping of waste by community members. Both the irregular collection of waste and insufficient supply of garbage bags are a problem of poor service delivery. All participants in this study revealed that these problems were caused by inadequate funding. The issue of inadequate funding is common in the service delivery literature in sub-Saharan Africa. Various authors [43, 44] explain that government taxies, usage fee revenues and aid are the main source of funding for water, sanitation and electricity in sub-Saharan Africa and yet the allocation of funding for these services is only 0.5% of the gross domestic product (GDP). In addition, some authors [45, 46] argue that municipalities also lack skilled people in the local government to run services delivery programmes adequately. The process of rolling out services to the communities is slow and hampers the quality and efficiency of waste management programmes [45, 46] Poor allocation of funding for waste management programmes could mean such services are not a priority to government. The government must promote sanitation programmes as one of the priority issues for protecting the health of its citizens by allocating adequate funding for waste management at the municipal level.

Our study reveals a lack of cooperation from community members in the removal of waste from homes and also during clean-up sessions in the community. The education officers revealed that they provide various education programmes in the communities and clean-up campaigns that aim at changing people’s attitudes towards waste management. Clean-up campaigns are really important and they give community members a sense of ownership not only of community goods but also of community problems. Clean up initiatives can also serve as deterrents to improper waste disposal. If participants know that they will be called out to clean up then they might be less likely to dispose waste improperly and also likely to discourage those who do so.

Research has shown that corruption is a persistent issue facing public service institutions in LMICs [47]. This study reveals allegations about corrupt tender processes for waste contractors thereby affecting service delivery. It is not clear how true these allegations are. However, a study on service delivery in South Africa found that most municipal officials in charge of awarding tenders were corrupt and were only interested in enriching themselves. Furthermore, the study revealed that policies on fighting corruption were not implemented and this led to misappropriation of funds among municipal officials without any accountability [48]. Considering that issues of corruption are much broader and cannot be addressed with one clear cut solution, we recommend that further studies must be conducted to provide in-depth insights into this issue.

Our study shows that incompetent contractors were hired to provide waste collection services in the communities and this undermined waste collection which had negative ramifications for the community as a whole. We recommend that further studies be conducted to explore this issue. The findings will inform efforts to solve the problem of corruption that are related to health care waste management.

The major strength of this study lies in its method. The qualitative approach illuminates how and why HCW is improperly managed in CBC. The policy-makers and stakeholders were the appropriate participants who provided insight into the issue of HCWM. The main limitation of the study was the fact that the perspectives of the people overseeing HCWM at the Department of Health were not explored. Their perspectives would have added more insight into waste management policies and practices at the level of the department.

## Conclusions

This study shows that the waste generated in community-based care is improperly managed. Given that South Africa has the highest HIV and TB prevalence in the world and majority of people living with HIV and TB receive care at home, it is imperative that policy-makers pay attention to HCWM in CBC. With the rollout of the new primary health care model, there is an even greater need to consider HCWM in CBC as a priority issue. Home-based care policies should be revised to include provisions for HCWM. Further research should be conducted with households and waste collectors to understand their HCWM experiences. Research could also be conducted with the Department of Health and other departments that have interest in HCWM issues to find out their perspectives about HCWM in homes. These studies could provide deeper insights into how HCW is managed from homes to the point of disposal. Finally, future research should seek to collect data that could be used to develop a conceptual framework that will help shed light on health care waste management in community-based care and further our understanding of this issue.

## Additional file

Additional file 1: Interview guides 1 to 3. The attached file contains 3 interview guides that consists of series of open-ended questions which were asked to all the participants that participated in this study. (DOCX 13 kb)

## Abbreviations

CBC: Community-based care
CBCs: Community-based care organisations
CBO: Community-based care
CHWs: Community health workers
DSW: Durban solid waste
HCW: Health care waste
HCWM: Health care waste management
HICs: High income countries
LMICs: Low-and-middle-income countries
PHC: Primary health care
RDP: Reconstruction and development programme
SANS: South African National Standards
TB: Tuberculosis
WHO: World Health Organisation
