# Supplementary material for: Perspectives of policy-makers and stakeholders about health care waste management in community-based care in South Africa: a qualitative study
Source: BMC Health Serv Res. 2017 Apr 19;17:290. doi: 10.1186/s12913-017-2236-x (PMC5395807; doi:10.1186/s12913-017-2236-x)
Supplement: Additional file 1: — Interview guides 1 to 3. The attached file contains 3 interview guides that consists of series of open-ended questions which were asked to all the participants that participated in this study. (DOCX 13 kb) [file 12913_2017_2236_MOESM1_ESM.docx]

**Additional file 1**

**INTERVIEW GUIDE 1: FOR WARD COUNCILLORS AND COMMUNITY-BASED CARE MANAGERS**

**DEMOGRAPHIC INFORMATION**

How long have you been working in the organisation?

What is your role in this community?

What are your duties in this community?

What are your major focus areas of development?

Please name the people that you work/collaborate with when doing your work.

What are the duties/roles of these people?

How many wards do you serve? Name the wards

How do you like your work?

**PERCEPTIONS AND EXPERIENCES REGARDING HEALTH CARE WASTE MANAGEMENT**

From your observation, how do people view health care waste management in the community?

Why do people see health care waste management that way?

What is your role with regards to health care waste management in this community?

**CHALLENGES REGARDING HEALTH CARE WASTE MANAGEMENT AND THEIR CAUSES**

From your observation and experience, what are the challenges that this community is having regarding health care waste management?

What could be the cause of these challenges?

**STRATEGIES USED TO DEAL WITH THE CHALLENGES FOR HEALTH CARE WASTE MANAGEMENT**

How do you deal with these challenges?

How do you feel about how these challenges are dealt with?

In your own opinion how best can these challenges be dealt with?

**INTERVIEW GUIDE 2 FOR THE AREA CLEANSING OFFICERS**

**DEMOGRAPHIC INFORMATION**

How long have you been working in the organisation?

What is your post in this organisation?

What does your job entail?

Please name the people that you work/collaborate with when doing your work.

What are the duties/roles of these people?

**PERCEPTIONS AND EXPERIENCES REGARDING HEALTH CARE WASTE MANAGEMENT**

How is health care waste handled in this community?

How often is health care waste collected from these communities?

What materials are provided for community members for use regarding health care waste management?

Who is responsible for providing these materials?

How often are these materials supplied and why?

How is health care waste separated within the homes?

What kind of guidelines do you use regarding management of health care waste?

Is it possible for us to have these copies?

What influence do you have in developing these rules/guidelines?

How and where can we get these rules and guidelines?

Who is in charge of developing these rules/guidelines?

How do you feel about these rules/guidelines?

How do these guidelines help you with your work and the community at large?

In your view, are these rules working?

What makes them work?

What is stopping them from working?

**CHALLENGES REGARDING HEALTH CARE WASTE MANAGEMENT AND THEIR CAUSES**

What challenges do the communities face while managing health care waste?

What could be the causes of these challenges regarding health care waste in these communities?

How do you deal with these challenges?

What do you think is the importance of handling health care waste properly?

What could be done to improve handling of health care waste in these communities?

**STRATEGIES USED TO DEAL WITH THE CHALLENGES FOR HEALTH CARE WASTE MANAGEMENT**

What efforts have you made to address the challenges regarding health care waste management in the communities?

What have been the successes of the efforts that you have made?

What challenges do you face while trying to make these efforts?

How do you deal with these challenges?

How do you feel about how these challenges are dealt with?

How would you like these challenges to be addressed?

**INTERVIEW GUIDE 3 FOR THE EDUCATION OFFICERS**

**DEMOGRAPHIC INFORMATION**

How long have you been working in the organisation?

How many wards do you serve? Name the wards

What is the role of your office or your position in this organisation?

What are your duties in this community?

Please name the people that you work/collaborate with when doing your work.

What are the duties/roles of these people?

**PERCEPTIONS AND EXPERIENCES REGARDING HEALTH CARE WASTE MANAGEMENT**

What kind of education do you offer with regards to health care waste management in the communities?

What materials do you use when conducting your education?

Who develops these materials?

What influence do you have in developing these materials?

Is it possible to have a copy of these materials?

From your observation and experience, how do people view health care waste management in the communities?

How do people react to the education that you provide about health care waste management?

How do you assess your education programmes?

**CHALLENGES REGARDING HEALTH CARE WASTE MANAGEMENT AND THEIR CAUSES**

What challenges do you face while doing your work in the communities?

What challenges does the community face regarding health care waste management?

What could be the causes of these challenges?

**STRATEGIES USED TO DEAL WITH THE CHALLENGES FOR HEALTH CARE WASTE MANAGEMENT**

How do you deal with these challenges?

Why do you deal with these challenges the way that you do?

What do you think could be the best way to deal with these challenges?
